# A non-invasive predictive model based on multimodality ultrasonography images to differentiate malignant from benign focal liver lesions

**DOI:** 10.1038/s41598-024-74740-7

**Published:** 2024-10-14

**Authors:** Qianqian Shen, Wei Wu, Ruining Wang, Jiaqi Zhang, Liping Liu

**Affiliations:** 1 Department of Medical Imaging, Shanxi Medical University, Taiyuan, Shanxi 030001 China; 2 Department of Ultrasound Intervention, First Hospital of Shanxi Medical University, Taiyuan, Shanxi 030001 China; 3 Department of Anorectal Surgery, Shanxi Provincial People’s Hospital, Taiyuan, Shanxi 030001 China; 4 Department of Ultrasound Intervention, First Hospital of Shanxi Medical University, Taiyuan, Shanxi 030001 China

**Keywords:** Sonazoid contrast-enhanced ultrasound, Sound touch elastography, Focal liver lesions, Nomogram, CEUS LI-RADS, Predictive model, Cancer, Medical research, Oncology, Imaging

## Abstract

We have developed a non-invasive predictive nomogram model that combines image features from Sonazoid contrast-enhanced ultrasound (SCEUS) and Sound touch elastography (STE) with clinical features for accurate differentiation of malignant from benign focal liver lesions (FLLs). This study ultimately encompassed 262 patients with FLLs from the First Hospital of Shanxi Medical University, covering the period from March 2020 to April 2023, and divided them into training set (*n* = 183) and test set (*n* = 79). Logistic regression analysis was used to identify independent indicators and develop a predictive model based on image features from SCEUS, STE, and clinical features. The area under the receiver operating characteristic (AUC) curve was determined to estimate the diagnostic performance of the nomogram with CEUS LI-RADS, and STE values. The C-index, calibration curve, and decision curve analysis (DCA) were further used for validation. Multivariate and LASSO logistic regression analyses identified that age, ALT, arterial phase hyperenhancement (APHE), enhancement level in the Kupffer phase, and Emean by STE were valuable predictors to distinguish malignant from benign lesions. The nomogram achieved AUCs of 0.988 and 0.978 in the training and test sets, respectively, outperforming the CEUS LI-RADS (0.754 and 0.824) and STE (0.909 and 0.923) alone. The C-index and calibration curve demonstrated that the nomogram offers high diagnostic accuracy with predicted values consistent with actual values. DCA indicated that the nomogram could increase the net benefit for patients. The predictive nomogram innovatively combining SCEUS, STE, and clinical features can effectively improve the diagnostic performance for focal liver lesions, which may help with individualized diagnosis and treatment in clinical practice.

## Introduction

Focal liver lesion (FLL) is a common disease of the liver, with the accurate differentiation between benign and malignant traits assuming paramount importance in treatment selection and patient prognosis. Conventional ultrasound, the primary tool for FLL screening, has inherent limitations in effectively discerning between benign and malignant lesions due to its low specificity^1^. Contrast-enhanced ultrasound (CEUS) is an accurate and convenient diagnostic method for liver lesions and has become an effective diagnostic imaging tool recommended in guidelines^2,3^. At present, the most widely used is the 2017 version of the CEUS Liver Imaging Report and Data System (CEUS LI-RADS) released by the American Radiological Society, mainly targeting HCC risk patients using pure blood pool contrast agents^4^. Nevertheless, conventional CEUS encounters challenges when confronted with highly differentiated malignancies, where the arterial phase (AP) may show high or equal enhancement, while the portal and delayed phases may lack the expected washout. This increases the risk of misclassifying malignant lesions as benign, leading to treatment delays and complicating prognostic assessments^5^. Consequently, there is an urgent need to develop a non-invasive diagnostic model with improved accuracy in distinguishing between benign and malignant liver lesions.

In recent years, Sonazoid contrast-enhanced ultrasound (SCEUS) and Sound touch elastography (STE) have captured growing attention due to their remarkable capabilities in discerning tumor blood perfusion and stiffness^6,7^. Sonazoid is a new liver-specific ultrasound contrast agent that not only has the same vascular phase as pure blood pool contrast agents but also has its independent late vascular phase (Kupffer phase, KP). Notably, during the KP imaging process, the reduced or absent presence of Kupffer cells in malignant lesions manifests as discernible defects, making KP defect analysis a robust diagnostic tool, exhibiting superior performance compared to CEUS LI-RADS, without compromising specificity^8,9^. Additionally, Sound touch elastography (STE) represents a recent breakthrough in elastography, grounded in the principles of 2D-shear wave elastography (2D-SWE). which can measure Young’s modulus of tissues^7^. Proliferation of connective tissue in malignant lesions can cause an increase in tissue elasticity. Previous studies have shown that measuring hardness using elastography can help distinguish benign and malignant of focal liver lesions^10–12^.

There are very few reports focusing on imaging features of SCEUS, STE, and clinical features for the diagnosis of FLLs. A nomogram is a graphical computational tool based on multiple regression analysis. It integrates multiple predictive indicators and transforms complex regression equations into simple and visual graphics, making the results of prediction models more readable and valuable. So we aim to establish a nomogram model based on combined image features of SCEUS, STE, and clinical features to improve the differential diagnosis ability of benign and malignant FLLs, thus helping with better decision-making for doctors in clinical practice.

## Methods

### Study population

**Figure 1:**
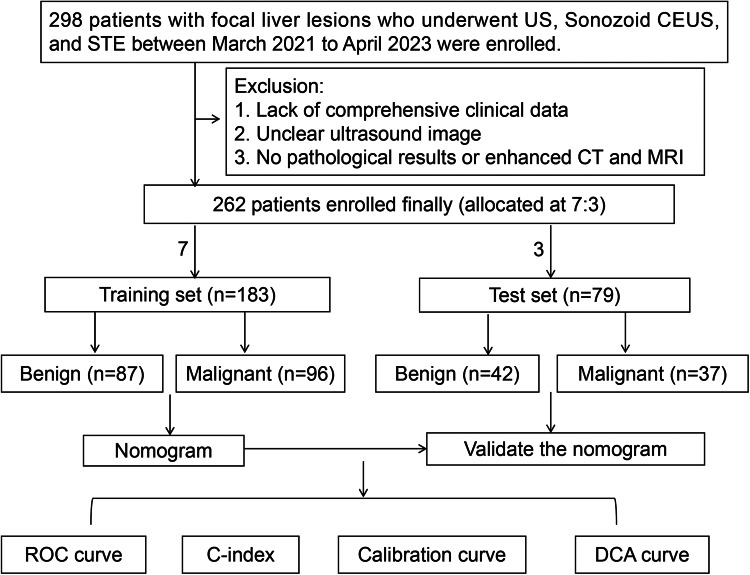
Flow chart of the study. CEUS: contrast-enhanced ultrasound; STE: Sound Touch Elastography; DCA: decision curve analysis.

The study was approved by the Ethics Committee of First Hospital of Shanxi Medical University (No. KYLL-2023-132). We finally enrolled 262 patients with FLLs who have undergone SCEUS and STE examinations. The malignant group and some benign lesions have been confirmed by surgical pathology or needle biopsy pathology, while the composite reference standard for some benign lesions was typical imaging features on the combination of enhanced magnetic resonance imaging(MRI) or enhanced computed tomography (CT) scans, and size stability and/or reduction during a minimum half a year follow-up period^9^. The exclusion criteria were as follows: (1) lack of comprehensive clinical data; (2) unclear ultrasound image; (3) no pathological results or enhanced CT and MRI. The clinic, imaging, and pathological data were organized for the enrolled participants. We divided the final enrolled patients into training set and validation set with a ratio of 7:3 according to the random generation number method. The flowchart of the study population is shown in Fig. 1.

### Clinical data collection

Clinical data including age, sex, body mass index **(**BMI), cirrhosis, hepatitis (hepatitis B and/or hepatitis C), alpha-fetoprotein (AFP), protein Induced by vitamin K absence or antagonist-II (PIVKA-II), carbohydrate antigen199 (CA199), carcinoembryonic antigen (CEA), platelet (PLT), glutamic-pyruvic transaminase (ALT), glutamic oxaloacetic transaminase (AST), total bilirubin (TBIL), direct bilirubin (DBIL), albumin (ALB), prealbumin (PA), total cholesterol (TC), prothrombin time (PT), prothrombin Time/international normalization ratio (PT/INR), and activated partial thromboplastin time (APTT).

### Ultrasound examination

**Figure 2:**
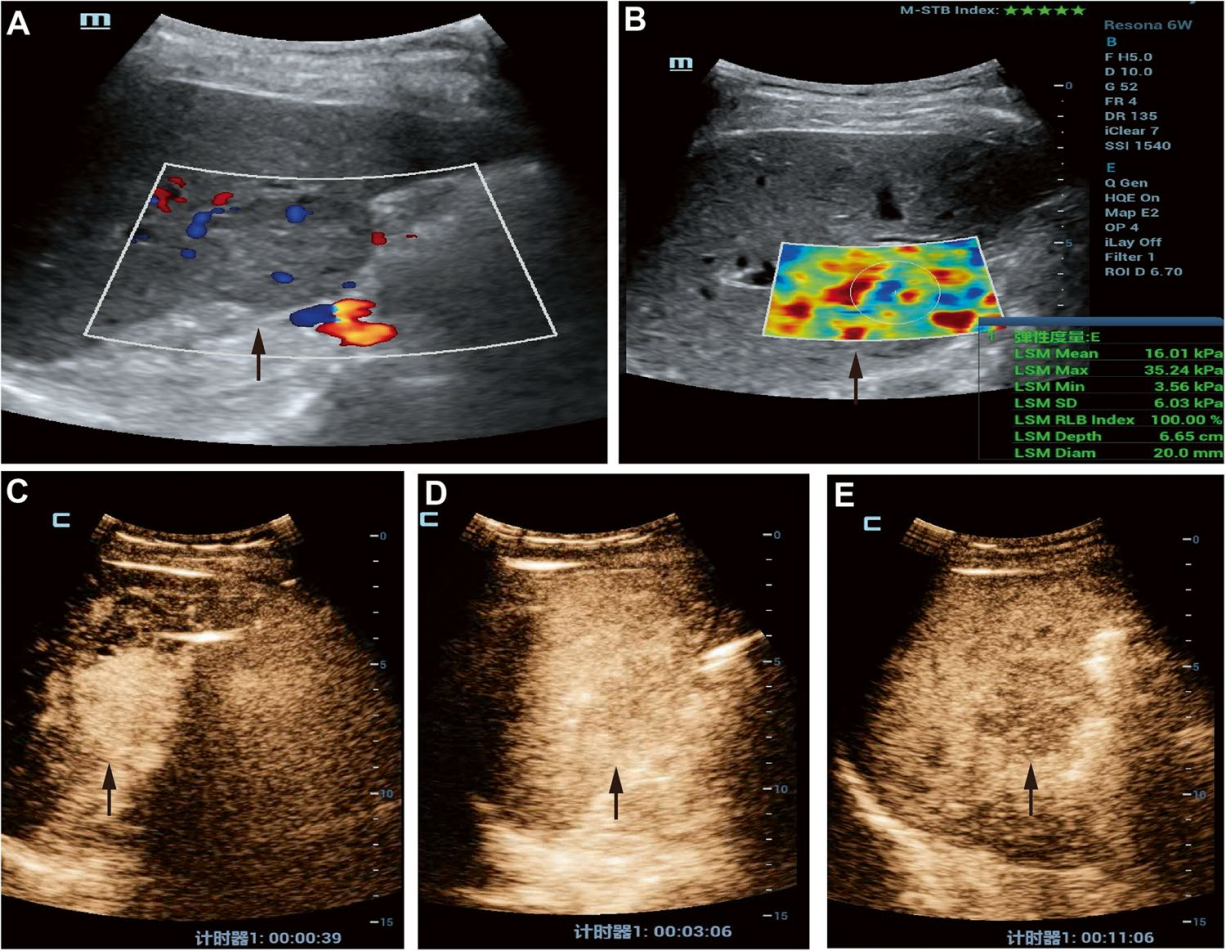
A 33-year-old HCC patient with an ALT of 23.4U/L. (**A**) Grayscale ultrasound shows hypoechoic lesions in the liver, with abundant blood flow and a maximum diameter of approximately 3.3 cm. Clear edges and regular shapes; (**B**) The STE display shows Emean as 16.01 kPa; (**C**) APHE appeared in the arterial phase after injection of Sonazoid contrast agent. (**D**) After 3 min, the lesion had no obvious washout; (**E**) After 10 min (Kupffer phase), the lesion showed low enhancement. According to CEUS LI-RADS, the lesion was considered benign. According to the column chart, the risk value is greater than 0.8); The lesion is predicted as malignant. Pathological diagnosis of highly differentiated HCC.

Routine ultrasound (Fig. 2A), STE (Fig. 2B), and SCEUS (Fig. 2C, D and E) examination all used Mindray (Shenzhen, China), Re6S color Doppler ultrasound diagnostic instrument, equipped with convex array broadband probe (SC6-1U). The patient lies on their back with the abdomen exposed and raises their right arm to fully expose the liver area. Observing the background of the liver and the location of the lesion, size, margin, internal echo, and blood flow of the lesions.

### Sound touch elastography examination

Choosing the elastography mode and adjusting the sampling box to include both the FLLs and some normal liver tissue around them. If FLLs are too big for the sampling box, we aim for non-necrotic areas and reselect the region to measure the liver tissue’s elasticity. The patient holds their breath while we observe the elastography color image. Once the image is stable for 3–5 s, we save it. repeat this process five times, and take the median. Each elasticity image shows a motion stability index, with four stars or more indicating good stability. For each measurement, the reliability index should be 90% or higher; otherwise, it’s considered invalid. See Fig. 2B for details.

### Sonazoid contrast-enhanced ultrasound examination

As shown in Fig. 2C, D and E, the second-generation contrast agent Sonazoid (GE Healthcare AS, Oslo, Norway) is a lipid-encapsulated perfluorobutane microbubble. Scaning the liver in two-dimensional ultrasound mode, find the optimal section to display the lesion, switch to contrast-enhanced ultrasound mode, instruct the patient to breathe calmly, and perform real-time dynamic imaging under the condition of mechanical index MI = 0.216. Injecting contrast agent (0.01 ml/kg) through the elbow vein using mass injection method, followed by 5 ml of physiological salt water, and start timing, videos of arterial phases, portal, and delayed phases (3–4 min after contrast injection) were stored. After vascular phases, the first 10s of intermittent imaging was stored every minute until 10 min or the disappearance of the liver parenchyma contrast agent (for evaluating Kupffer phase). All the images were stored on hard drives for subsequent analysis.

### Ultrasound image analysis

Ultrasound images were retrospectively reviewed by two sonographers who had more than ten years of experience in abdominal ultrasound imaging, independently. On SCEUS images, the following individual features of each target observation were reviewed: (1) With or without APHE, (2) With or without washout, and (3) the presence of Kupffer hypoenhancement or defect, (4) Is the enhanced morphology regular, (5) Whether the margin is clear after enhancement was also assessed. The assessments of APHE and washout patterns were based on criteria in CEUS LI-RADS: tumors with APHE and washout in a portal or delayed phase were diagnosed as CEUS LI-RADS malignant lesions; others were considered CEUS LI-RADS benign lesions. Observations were considered to exhibit a Kupffer hypoenhancement or defect when showing hypoechogenicity relative to liver parenchyma in the Kupffer phase.

### Development and validation of a nomogram to distinguish malignant from benign lesions

Firstly, potential risk factors are identified through univariate logistic regression in the training set. Afterward, with the variables with *p* < 0.05 in univariate regression, redundant and irrelevant variables were removed through LASSO regression to reduce model complexity. Finally, variables with LASSO regression coefficient > 0 were included in the multivariate logistic regression to screen for independent risk factors (*p* < 0.05) and a nomogram model was established. To assess the effectiveness of the nomogram, we compared the performance of three methods: nomogram, CEUS LI-RADS, and STE. Receiver operator characteristic (ROC) curves were generated for three methods in both the training set and test set and the area under the curves (AUC) was calculated to assess the diagnostic value of the nomogram. DeLong’s test was used to compare the performance of the nomogram, CEUS LI-RADS, and STE alone. The concordance index (C-index), and the calibration curve were used to evaluate the predictive accuracy and consistency of the three methods. Decision curve analysis (DCA) was conducted by quantifying the net benefits at different threshold probabilities, to evaluate the clinical usefulness of the three methods above in both training and test sets.

### Statistical analysis

All analyses were performed using SPSS 25.0 (IBM Corp, Armonk, NY, USA), MedCalc software (Mariakerke, Belgium), and R 4.3.2 software. The differences in SCEUS, STE, and clinical features were compared between the training set and test set using independent sample t-tests, and chi-square tests. Univariate and multivariate regression analyses were performed via SPSS 25.0. Via R software, the “glmnet” package was used for LASSO regression, the “rms” package was used to plot the nomogram, the " ggstatspot” package was used to construct the calibration curves, the “rmda” package was used for the decision curves analysis. ROC curve analysis and McNemar test were drawn via MedCalc. The difference was statistically significant when *p* < 0.05.

## Results

### Patient characteristics

As shown in Fig. 1, a total of 298 consecutive patients, after screening for study inclusion and exclusion criteria, a total of 262 FLL patients (162 male patients, 100 female patients) from the First Hospital of Shanxi Medical University were enrolled finally, including 124 malignant liver cancer patients, including hepatocellular carcinoma (HCC) (*n* = 113); intrahepatic cholangiocarcinoma (ICC) (*n* = 4); liver metastases (*n* = 4); hepatic lymphoma (*n* = 3). 138 benign liver cancer patients, including hemangioma (*n* = 85), adenomas (*n* = 17), focal nodular hyperplasia (FNH) (*n* = 23), regenerate nodules in liver cirrhosis (*n* = 6), inflammatory pseudo-tumor (*n* = 7). There were 183 patients in the training set, consisting of 87 with malignant lesions and 96 with benign lesions. And 79 patients in the test set which consist of 37 malignant lesions and 42 benign lesions. The baseline characteristics of the SCEUS, STE, and clinical features are summarized and compared between training and test sets in Table 1. There was no significant difference between the two sets of patients, which justified the grouping of training and test sets.

**Table 1:** Image features of SCEUS, STE, and clinical features of patients.

Characteristics	Training set	Test set	*p*-value
	(*n* = 183)	(*n* = 79)	
**Clinical feature**			
Age (years)			0.744
<40	23 (12.6%)	7 (8.9%)	
≥40	160 (87.4%)	72 (91.1%)	
Sex			0.052
Female	65 (35.5%)	28 (35.4%)	
Male	118 (64.5%)	51 (64.6%)	
BMI (kg/m^2^)	20.97 ± 2.87	21.95 ± 3.37	0.159
Hepatitis			0.133
Negative	81 (44.3%)	46 (58.2%)	
Positive	102 (55.7%)	33 (41.8%)	
Cirrhosis			0.284
Negative	75 (40.9%)	52 (65.8%)	
Positive	108 (59.1%)	27 (34.2%)	
AFP			0.312
<20ug/L	132 (72.1%)	63 (79.7%)	
≥20ug/L	51 (27.9%)	16 (20.3%)	
CEA (ug/L)	44.68 ± 34.91	2.65 ± 2.39	0.280
CA199			0.312
<37u/mL	148 (80.9%)	70 (88.6%)	
≥37u/mL	35 (19.1%)	9 (11.4%)	
PLT (109/L)	140.65 ± 58.54	150.73 ± 61.22	0.208
ALT (U/L)	65.99 ± 63.44	46.05 ± 92.71	0.217
AST (U/L)	40.69 ± 62.88	20.97 ± 27.05	0.054
TBIL (µmol/L)	37.28 ± 61.73	53.19 ± 49.79	0.548
DBIL (µmol/L)	12.63 ± 36.18	9.85 ± 29.41	0.313
ALB (g/L)	36.331 ± 7.35	35.40 ± 5.39	0.464
TC (mmol/L)	4.06 ± 1.54	3.90 ± 1.63	0.475
PTS (s)	21.68 ± 6.10	15.37 ± 3.04	0.561
APTT (s)	32.92 ± 9.39	32.76 ± 8.71	0.895
**Sonozoid**			
Tumor size			0.596
<2 cm	39 (11.3%)	4 (5.1%)	
≥2 cm	144 (78.7%)	75 (94.9%)	
Morphology			0.473
Regular	152 (83.1%)	69 (87.3%)	
Irregular	31 (16.9%)	10 (12.7%)	
Margin			0.545
Clear	149 (81.4%)	65 (82.3%)	
Unclear	34 (18.6%)	14 (17.7%)	
APHE			0.122
Negative	67 (36.6%)	37 (46.8%)	
Positive	116 (63.4%)	42 (53.2%)	
Washout			0.686
Negative	65 (35.5%)	26 (32.9%)	
Positive	118 (64.5%)	53 (67.1%)	
Kupffer			0.772
Iso or hyperenhancement	80 (43.4%)	33 (41.8%)	
Hypoenhancement or defect	104 (56.6%)	46 (58.2%)	
**STE**			
Emax (kPa)	23.97 ± 5.77	24.66 ± 7.62	0.649
Emean (kPa)	13.01 ± 5.36	12.33 ± 6.67	0.136

### Identify independent variables significantly associated with malignant lesions

**Figure 3:**
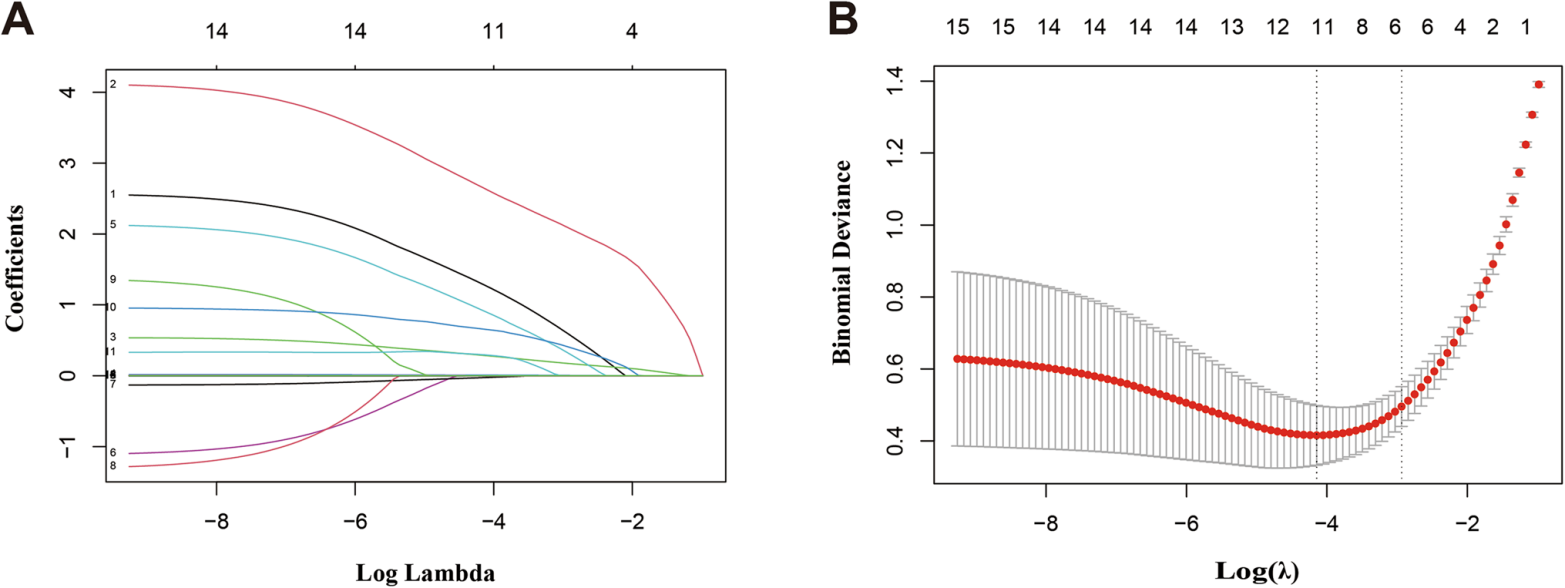
SCEUS, STE, and clinical feature selection with least absolute shrinkage and selection operator (LASSO) regression in the training set. (**A**) A coefficient profile plot was produced against the log(lambda) sequence; (**B**) The value of l that gave the minimum average binomial deviance was used to select features. Dotted vertical lines were drawn at the optimal values using the minimum criteria and the 1-SE criteria.

With the image features of SCEUS, STE, and clinical features, univariate logistic regression was compared between malignant and benign lesions in the training set. After the screening, 15 variables including Age, BMI, Hepatitis, Cirrhosis, AFP, CEA, CA199, PLT, ALT, AST, PTS, APHE, Washout, Kupffer, Emean with statistical significance (*p* < 0.05) (Table 2). Given a large number of variables, LASSO regression was applied to solve the multicollinearity relationships of all features, and the coefficients of each variable were generated to further screen factors from the univariate analysis result. The non-zero coefficients were considered to have strong prognostic potential in the LASSO penalized regression model. As a result, a total of 11 key variables including Age, Hepatitis, AFP, CA199, CEA, ALT, AST, APHE, Washout, Kupffer, and Emean were left for the LASSO regression (Table 3; Fig. 3).

**Table 2:** Univariate and multivariate analysis of SCEUS, STE, and clinical features.

Variables	Univariate analysis				Multivariate analysis		
	B	OR	95%CI	*p*	OR	95%CI	*p*
**Clinical features**							
Age (≥ 40 years)	2.058	7.83	3.704–16.554	< 0.001	12.491	1.962–79.511	0.0075
BMI (kg/m^2^)	0.309	0.734	0.646–0.834	< 0.001			
Hepatitis	1.755	5.786	2.893–11.571	< 0.001			
Cirrhosis	0.677	2.404	1.259–4.588	0.008			
AFP (≥ 20ug/L)	3.642	38.154	11.209-129.866	< 0.001			
CEA (ug/L)	0.12	1.127	1.010–1.257	0.032			
CA199 (≥ 37u/mL)	1.599	4.95	2.106–11.633	< 0.001			
PLT (10^9^/L)	0.007	0.993	0.987–0.998	0.007			
ALT (U/L)	0.025	1.025	1.016–1.035	< 0.001	1.017	1.003–1.031	0.016
AST (U/L)	0.011	1.011	1.002–1.020	0.02			
PTS	0.207	1.112	1.084–1.396	0.001			
**SCEUS**							
APHE	2.169	8.75	4.208–18.195	< 0.001	137.599	6.452-2934.673	0.0016
Washout	2.696	14.781	6.731–32.458	< 0.001			
Kupffer	3.967	52.8	21.193-131.546	< 0.001	64.508	8.465-491.578	0.0001
**STE**							
Emean (kPa)	0.463	1.588	1.397–1.806	< 0.001	1.682	1.104–2.562	0.0156

**Table 3:** Results of LASSO regression analysis.

Variables	LASSO regression coefcient
Age (≥ 40 years)	1.531136158
Hepatitis	0.128592573
ALT (U/L)	0.009622183
AST (U/L)	0.000376831
AFP (≥ 20ug/L)	0.900381271
CA199 (≥ 37u/mL)	0.299144702
CEA (ug/L)	0.00030138
APHE	1.531136158
Washout	0.255025945
Kupffer	2.576129498
STEmean (kPa)	0.177796721

### Development and validation of the nomogram

**Figure 4:**
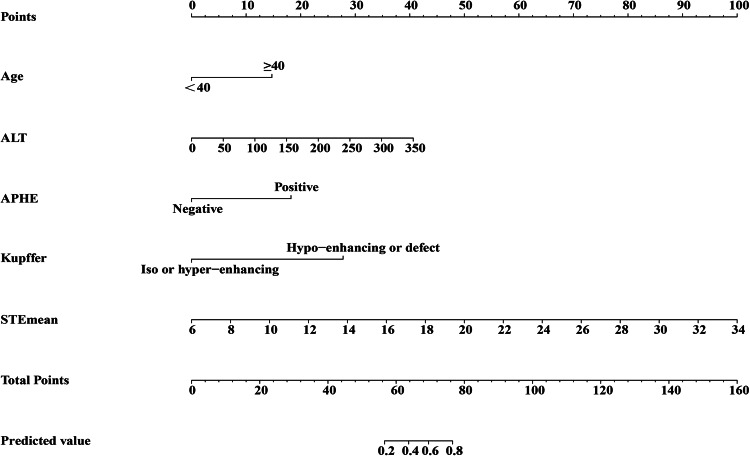
Nomogram incorporating image features of SCEUS, STE, and clinical features for distinguishing malignant lesions from benign lesions.

To further evaluate the combination effects of multiple factors, the final model includes 5 variables according to the multivariate logistic regression analysis with *p* < 0.05. Predicted value = − 12.237 + 2.048 Age + 0.0161 ALT + 2.534 APHE + 3.862 Kupffer + 0.496 Emean. As shown in Fig. 4, the nomogram established based on Age, ALT, APHE, Kupffer, and Emean, the probability of malignant lesions could be estimated for each patient. The C-index of the nomogram was 0.988.

As shown in Fig. 6; Table 4. The AUC in the training set and test set were 0.988 (95% CI 0.963–0.999) and 0.978 (95% CI 0.917–0.998), which was higher than CEUS LI-RADS (0.754 and 0.824 ) and STE (0.909 and 0.923) (all *p* < 0.001). The AUC of the nomogram model was significantly higher than that of CEUS LI-RADS in both training and test sets (Z = 7.714 and 3.929, *p* ≤ 0.0001), but was not significantly different from that of STE in test set (Z = 1.931, *p* = 0.0534). The nomogram achieved sensitivity, specificity, LR+, and LR- of 95.40%, 95.83%, 22.9, and 0.048 in training set and 94.59%, 95.24%, 19.86, and 0.057 in test set, respectively.

**Table 4:** Diagnostic efficacy of different methods in training set and test set.

Set	Methods	AUC (95% CI)	*P* value	Cut-off	Sensitivity	Specificity	LR+	LR-
Training set	CEUS LI_RADS	0.754 (0.685–0.815)	< 0.0001	0.272	64.37%	86.46%	4.75	0.41
	STE	0.909 (0.857–0.946)	< 0.0001	0.414	81.61%	88.54%	7.12	0.21
	Nomogram	0.988(0.963–0.999)	< 0.0001	0.548	95.40%	95.83%	22.9	0.048
Test set	CEUS LI_RADS	0.824 (0.722–0.901)	< 0.0001	0.236	64.86%	100%	—	0.35
	STE	0.923 (0.840–0.971)	< 0.0001	0.260	86.49%	90.48	9.08	0.15
	Nomogram	0.978 (0.917–0.998)	< 0.0001	0.299	94.59	95.24	19.86	0.057

**Figure 5:**
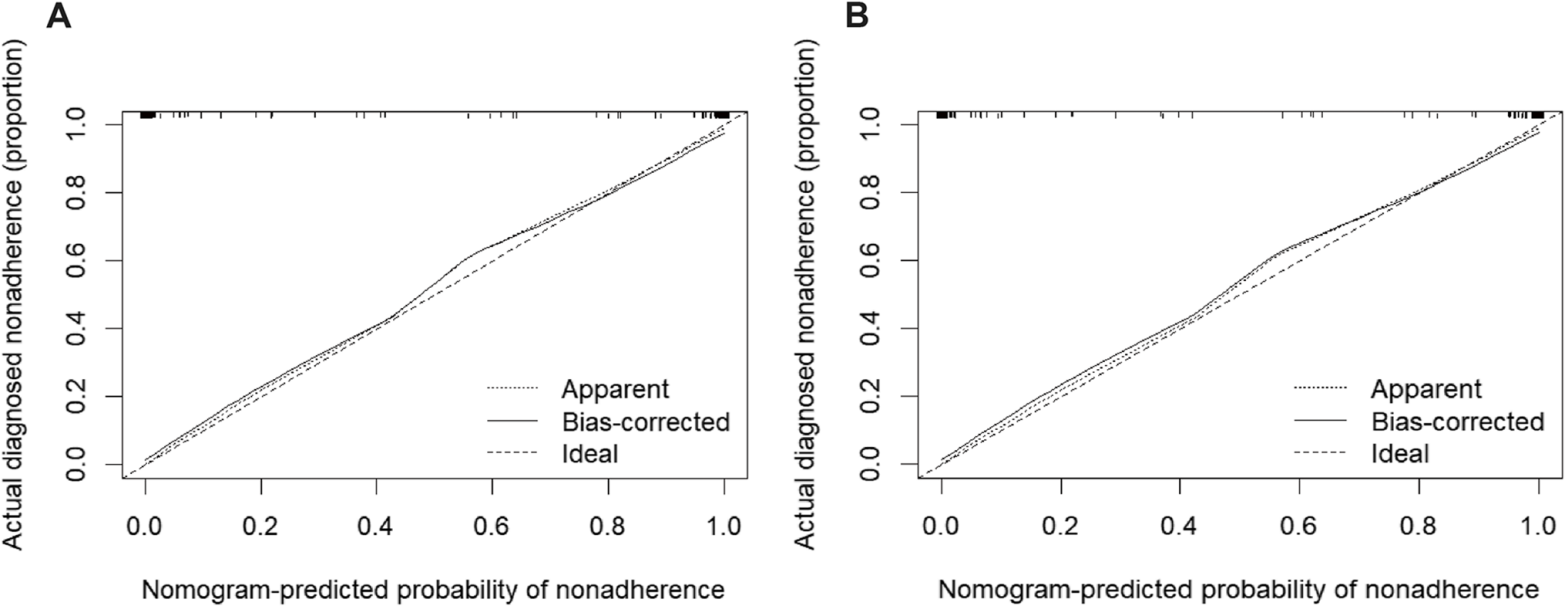
Calibration curves of the nomogram prediction. The y-axis indicates the actual diagnosed ICC. The x-axis indicates the predicted risk of ICC. The diagonal dotted line indicates a perfect prediction by an ideal model. The solid line represents the performance of the cohort, which indicates that a closer fit to the diagonal dotted line represents a better prediction.

**Figure 6:**
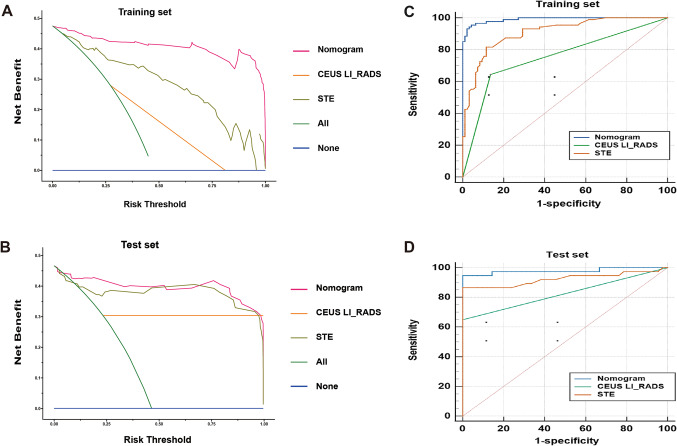
Performance and clinical usefulness evaluation of nomograms. (**A**, **B**): Analysis of DCA derived from the training set and validation set concluded the use of nomogram provides more benefits for patients than CEUS LI-RADS or STE diagnosis alone. (**C**, **D**): The AUCs obtained from the training set and validation set have better performance than the CEUS LI-RADS and STE alone.

The calibration curve (Fig. 5) demonstrated that there was a good agreement between the actual observations and predicted probabilities of the nomogram. Furthermore, The DCA curve compared the clinical usefulness of three methods. The clinical net benefit of the nomogram is higher than that of CEUS LI-RADS and STE alone in both the training and test sets (Fig. 6).

## Discussion

In this study, we developed a robust predictive nomogram model integrating Sonazoid contrast-enhanced ultrasound (SCEUS), Sound Touch Elastography (STE), and clinical variables, employing a comprehensive analytical approach encompassing univariate, LASSO, and multivariate logistic regression analyses. This nomogram is the first study to combine image features of SCEUS (specific Kupffer phase), STE (2D-SWE), with clinical features for discriminating between benign and malignant focal liver lesions. Notably, our findings revealed that the nomogram model of multimodal ultrasound nomogram demonstrated heightened diagnosis performance (0.988 and 9.978 in training and test sets) in detecting malignant lesions, outperforming both CEUS LI-RADS and STE alone. Compared with previous studies which mostly use Sonovue contrast-enhanced ultrasound or elastography alone to diagnose benign and malignant liver diseases (with AUC ranging from 0.813 to 0.942)^13–15^, our nomogram also demonstrated excellent diagnosis. Firstly, our model fully considers the hardness of the lesion, blood perfusion, and blood biochemical indicators, and evaluates the lesion from multiple perspectives, which can effectively reduce missed diagnoses and misdiagnosis caused by single feature evaluation. Meanwhile, we used the latest liver-specific ultrasound contrast agent Sonozoid, which has a specific Kupffer phase compared to Sonove, as Kupffer phase contrast agent hypoenhancement or defect is the most important feature of malignant tumors. Moreover, DCA highlighted the added clinical utility of integrating SCEUS with STE measurements, surpassing the efficacy of either modality in isolation, thereby enhancing the management of FLL patients.

APHE, enhancement levels during the KP, and Emean by STE were identified as independent risk factors crucial for distinguishing between malignant and benign lesions. Our findings showed that malignant lesions exhibited a notably higher proportion of APHE compared to benign lesions, a trend consistent with previous researches^16–18^. The presence of heightened enhancement during the arterial phase signifies robust blood flow within the nodule. A prospective multicenter study corroborated this observation, revealing that 85.8% of HCCs demonstrated APHE, with an impressive positive predictive value for malignancy of 94.6% among patients at risk for HCC^18^. In our study, Most malignant tumors in the Kupffer phase showed obvious hypoenhancement or defects while benign tumors did not, thus indicating that the Kupffer phase showed high specificity in the diagnosis of malignant lesions. Importantly, these observations align closely with findings from prior studies^9,19,20^. This is because there are rare or no Kupffer cells in malignant tumors, thereby manifesting as hypoenhancement or defect during the Kupffer phase^21,22^. Additionally, In addition, previous studies have suggested that 10–33% of HCC exhibit washout during the Kupffer phase, but not during the vascular phase^23,24^, this observation suggests that the decline in Kupffer cell density may precede alterations in portal flow within the tumor during HCC evolution, thereby enhancing the diagnostic sensitivity of Sonazoid in identifying HCCs. A prospective study comparing Sonazoid and pure blood-pool CEUS within the framework of CEUS LI-RADS diagnostic criteria—specifically, arterial phase hyperenhancement (APHE) and mild washout in kupffer phase for HCC—demonstrated a significantly heightened sensitivity for Sonazoid CEUS (79%) compared to pure blood-pool CEUS (54%), while maintaining equivalent specificity (100%)^9^. Therefore, using Kupffer phase washout as the main imaging feature of SCEUS can improve the diagnostic efficiency of benign and malignant liver diseases. In our study, the utilization of STE yielded noteworthy results in distinguishing between benign and malignant lesions. Specifically, The Emean values of malignant lesions significantly exceeded those of benign lesions, consistent with prior research findings, with AUCs reaching 0.9^15,25,26^. Malignant lesions, characterized by dense cancerous nests and invasive behavior in surrounding tissues, inherently exhibit higher Young’s modulus values compared to benign lesions and normal liver tissue^27^. Thus, the integration of APHE, Kupffer phase characteristics, and Emean values into our diagnostic model effectively enhances the discrimination between benign and malignant lesions.

We also included ALT and age as clinical features in the model, and when these variables were included, the net benefit increased significantly. ALT is closely related to hepatocellular injury, and the presence of liver cancer is usually accompanied by severe hepatocellular injury, coupled with the frequent coexistence of chronic hepatitis and cirrhosis in patients, which together lead to the elevation of ALT levels. Yan Du et al. concluded that elevated ALT levels serve as a robust predictor of HCC, accentuating the heightened risk of HCC development associated with elevated ALT levels^28^. Age, is another key variable in our nomogram, aligning closely with clinical and epidemiological evidence regarding HCC^29^. Recommendations from the American Association for the Study of Liver Diseases (AASLD) advocate for the regular cancer screening of chronic hepatitis B patients aged 40 and above, emphasizing the critical importance of age in timely HCC detection and intervention^30^. Therefore, the inclusion of ALT and age in our model has important clinical significance in differentiating benign and malignant liver lesions.

In the assessment using CEUS LI-RADS, there were notable instances of misdiagnosis, including one case of adenomas hepatocellular adenoma, two cases of inflammatory pseudo-tumor and two cases of highly differentiated liver cancer, but the nomogram provided correct diagnoses. We attribute these discrepancies to the unique characteristics of certain adenomas and inflammatory pseudo-tumors, which can exhibit abundant blood flow and demonstrate APHE and subsequent washout in the portal or delayed phase^31–33^. Consequently, CEUS LI-RADS may misclassify them as malignant, whereas the nomogram, integrating Kupffer phase data, Emean values, and clinical indicators, offers a more accurate diagnosis, thereby reducing the misdiagnosis rate. Moreover, highly differentiated liver cancers typically manifest APHE without washout in the portal and delayed phases^34,35^, a feature that may lead to misclassification as benign lesions by CEUS LI-RADS. But nomogram can be diagnosed as malignant according to its low enhancement in Kupffer phase and high Emean of STE. In our evaluation of FLLs using STE, two cases of FNH were erroneously diagnosed as malignant, which can be attributed to the greater proportion of fibrotic or scar tissue in FNHs, leading to increased tissue hardness^25,36,37^, The nomogram, by integrating blood flow information and considering the absence of Kupffer phase low enhancement or defects characteristic of FNH, enhances diagnostic accuracy and reduces misdiagnosis rates. Therefore, the nomogram emerges as a valuable tool in distinguishing benign and malignant FLLs, thereby facilitating correct clinical decision-making.

This study has several limitations. Firstly, this is a single-center retrospective study, and in the future, our conclusions should be validated through multicenter prospective studies. Secondly, our sample size is relatively small, and in the future, we should increase the sample size to better train the nomogram model. Thirdly, the nomogram model of this study can effectively distinguish between benign and malignant liver nodules, but the classification of different types of malignant nodules is not yet clear. This means that the model may perform well in a specific patient population, but its predictive performance may be compromised when applied to a completely different patient population. And for some special cases or complex cases, the nomogram diagnostic model may not provide accurate predictions. Our research group has been dedicated to the ultrasound diagnosis of liver cancer and will further collect data in the future, striving to establish diagnostic models for different types of malignant nodules to better guide prognosis.

## Conclusion

An effective predictive nomogram innovatively combining SCEUS, STE, and clinical features can improve the diagnostic performance of benign and malignant liver nodules. Its diagnostic efficacy is higher than that of CEUS LI-RADS and STE alone, which contributes to personalized diagnosis and precision medicine in clinical practice, thereby improving the prognosis of malignant tumors. I hope that in future research, prospective multicenter large sample analysis can be conducted to effectively and accurately distinguish between benign and malignant liver lesions and diagnostic models should be established by collecting different types of liver lesions to better classify malignant tumors.

## Data Availability

The datasets used and/or analysed during the current study are available from the corresponding author on reasonable request.

## Abbreviations

SCEUS: Sonazoid contrast-enhanced ultrasound
STE: Sound Touch Elastography
FLLs: focal liver lesions
ROC: Receiver Operating Characteristic Curves
AUC: Areas Under the Receiver Operating Characteristics Curves
DCA: decision curve analysis
CEUS LI-RADS: CEUS Liver Imaging Report, and Data System
AP: arterial phase
KP: Kupffer phase
SWE: shear wave elastography
BMI: body mass index
AFP: alpha-fetoprotein
PIVKA-II: proteinInduced by vitamin K absence or antagonist-II
CA199: carbohydrate antigen199
CEA: carcinoembryonic antigen
PLT: platelet
ALT: glutamic-pyruvic transaminase
AST: glutamic oxaloacetic transaminase
TBIL: total bilirubin
DBIL: direct bilirubin
ALB: albumin
PA: prealbumin
TC: total cholesterol
PT: prothrombin time
PT/INR: prothrombin Time/international normalization ratio
APTT: activated partial thromboplastin time
APHE: arterial phase hyperenhancement pattern
C-index: concordance index
HCC: hepatocellular carcinoma
FNH: focal nodular hyperplasia
